# Immunotherapy in Hepatocellular Carcinoma

**DOI:** 10.1007/s11864-021-00886-5

**Published:** 2021-08-23

**Authors:** Claudia A. M. Fulgenzi, Thomas Talbot, Sam M. Murray, Marianna Silletta, Bruno Vincenzi, Alessio Cortellini, David J. Pinato

**Affiliations:** 1 Division of Medical Oncology, Policlinico Universitario Campus Bio-Medico, Rome, Italy; 2 Department of Surgery & Cancer, Hammersmith Hospital, Imperial College London, Hammersmith Campus, Du Cane Road, W120HS, London, UK; 3 Department of Infectious Disease, Faculty of Medicine, Hammersmith Hospital, Imperial College London, W12 0NN, London, UK; 4 Department of Biotechnology and Applied Clinical Sciences, University of L’Aquila, Via Vetoio, 67100 L’Aquila, Italy; 5 Division of Oncology, Department of Translational Medicine, University of Piemonte Orientale, Novara, Italy

**Keywords:** HCC, Immunotherapy, PD-1, CTLA-4, VEGF

## Abstract

Patients with hepatocellular carcinoma (HCC) have been traditionally deprived from highly effective systemic therapy options in the past decades. The multi-targeted tyrosine kinase inhibitor sorafenib, approved in 2008, remained the only treatment option for advanced HCC for over a decade. A number of molecularly targeted therapies such as lenvatinib, regorafenib, cabozantinib, and ramucirumab have significantly widened treatment options in patients with advanced HCC. However, emergence of resistance and long-term toxicity from treatment are barriers to long-term survivorship. Immunotherapy is at the focus of intense research efforts in HCC. Whilst targeting of programmed cell death 1 (PD-1) and cytotoxic T lymphocyte 4 (CTLA-4) is associated with radiologically measurable disease-modulating effects in HCC, monotherapies fell short of demonstrating evidence of significant survival extension in advanced disease. Atezolizumab and bevacizumab were the first immunotherapy regimen to demonstrate clear superiority in improving the survival of patients with unresectable HCC compared to sorafenib, paving the way for immunotherapy combinations. As the treatment landscape of HCC rapidly evolves, with immunotherapy integrating within early- and intermediate-stage disease treatment algorithms, lack of level 1 evidence on sequencing of therapeutic strategies and lack of head-to-head comparisons across immunotherapy combinations will affect prescribing of immunotherapy in routine practice. In the absence of predictive biomarkers, choice of immunotherapy over kinase inhibitors will continue to remain an empirical exercise, guided by balancing anti-tumour efficacy with toxicity considerations in the individual patient.

## Introduction

Immunotherapy is gaining continued traction in treatment of different types of cancers. Immune checkpoint molecules are central in maintaining immune tolerance and programmed cell death 1 (PD-1) and cytotoxic-T-lymphocyte-associated protein 4 (CTLA-4) have strongly emerged in immuno-oncology for their role as therapeutically actionable drivers of immune escape [1]. The interaction between PD-1, CTLA-4, and their ligands (PD-L1/2 and B7-1/2) inhibits T-cell activation [2]. In recent years, immune checkpoint inhibition has emerged as an efficacious anti-cancer strategy, with several anti PD-1, anti PD-L1, and anti CTLA-4 antibodies being approved for treatment of various malignancies [3]. Nivolumab, pembrolizumab, ipilimumab, tremelimumab, atezolizumab, and durvalumab are among the immune checkpoint inhibitors (ICI) with demonstrated efficacy active in hepatocellular carcinoma (HCC) [4].

The liver hosts an immunosuppressive microenvironment, constantly receiving many different antigens from the gut [2]. HCC usually develops in the context of chronic inflammation and cirrhosis, a primary cause of immune exhaustion that, in turn, enhances liver immunosuppressive status [5]. PD-L1 overexpression in cancer cells and in local antigen-presenting cells (APCs) is common in HCC and it has a recognized negative prognostic value [6]. These considerations infer a strong therapeutic rationale for immune checkpoint inhibition in treatment of HCC [2]. The safety of immune checkpoint inhibitors in patients with underlying liver disease has been of concern for many years. Historically, patients affected by viral hepatitis or liver disfunction have been excluded by immune checkpoint inhibitors trials. However, recent clinical trials have demonstrated the feasibility of this approach [7], paving the way for the development of immunotherapy in HCC.

## PD-1/PD-L1 inhibitors

Nivolumab is a fully humanized IgG4 monoclonal antibody directed against PD-1, preventing its interaction with PD-1 ligands thereby restoring immune activity against cancer cells [8]. In 2017, the US Food and Drug Administration (FDA) granted breakthrough approval for advanced HCC after sorafenib failure [9] based on the results of CheckMate-040 [8]. The study enrolled 48 patients in the dose-escalation and 214 in the dose-expansion phases: 182 of 262 (69.5%) were sorafenib-pre-treated. In the dose-expansion group, nivolumab at 3 mg/kg every 2 weeks led to an objective response rate (ORR) of 20% by Response Evaluation Criteria in Solid Tumours (RECIST) 1.1 criteria. Updated results revealed an ORR of 15% and a median overall survival (OS) of 15.1 months for sorafenib-experienced patients [10].

Following the encouraging results of CheckMate-040, the efficacy of front-line nivolumab was compared to standard of care sorafenib in advanced HCC in the phase III CheckMate-459 study [11, 12]. OS was elected as primary endpoint, with a hazard ratio (HR) of 0.84 in favour of nivolumab chosen as the pre-specified threshold for statistical significance. Secondary endpoints included ORR; progression-free survival (PFS) according to RECIST v.1.1 criteria; safety; and efficacy, stratified by PD-L1 expression.

Although nivolumab yielded the longest median OS seen in first-line advanced HCC trials at the time of reporting, CheckMate-459 did not meet its primary endpoint, with median OS of 16.4 months for nivolumab and 14.7 months for sorafenib, with a HR of 0.85 (95% confidence interval (CI): 0.72–1.02; p = 0.0752).

OS in the sorafenib arm was also strikingly higher than observed in the Sharp and Asia-Pacific trials as well as in historical cohorts [13]. This may be explained by greater experience in managing sorafenib AEs and availability of second-line agents [1]. ORR according to the RECIST 1.1 criteria by blinded independent central review was 15% for nivolumab and 7% for sorafenib. Patients with tumoural PD-L1 expression ≥ 1% on IHC had an ORR of 28% (20/71) compared to 12% (36/295) in case of PD-L1 < 1%; statistical significance of this higher ORR was not assessed. CheckMate-459 confirmed the overall safety of nivolumab with grade 3–4 AEs being reported in 22% of patients in the experimental arm and 49% in sorafenib arm.

Pembrolizumab is another fully humanized monoclonal antibody against PD-1. It was approved by the FDA for advanced HCC after sorafenib failure or intolerance in 2018. Safety and activity were tested in an open-label phase II trial: KEYNOTE-224 [14], and efficacy was then evaluated in a phase III study: KEYNOTE-240 [15]. KEYNOTE-224 assessed the efficacy of pembrolizumab (200 mg every 3 weeks) in 104 patients that had progressed (80%) or were intolerant (20%) to sorafenib. PD-L1 expression was scored by combined positive score (CPS) and tumour proportion score (TPS) [16]. ORR in the intention to treat (ITT) population was of 17%, median PFS was 4.9 months (95% CI 3.4–7.2), and median OS was 12.9 months (95% CI, 9.7–15.5). Fifteen (16%) patients experienced serious treatment-related adverse events (TRAE). Exploratory analysis of the predictive role of PD-L1 expression was conducted from data available for 52 patients. Overall, 22 out of 52 subjects (42%) were CPS-positive and 7 (13%) were TPS-positive. ORR was 32% for CPS-positive patients vs. 20% for those with negative CPS, whilst ORR was 43% for patients with TPS ≥ 1% and 22% for those with TPS < 1%. The predictive role of PD-L1 expression here is however limited by the retrospective nature of the analysis and overall paucity of PD-L1-assessed patients.

The efficacy of pembrolizumab after sorafenib was further tested in KEYNOTE-240, a randomized phase III, placebo-controlled, double-blind trial [15], which evaluated 443 patients randomized at a 2:1 ratio to receive pembrolizumab (278) or placebo (135). Median OS was 13.9 months for the experimental arm, 10.6 months for placebo (HR 0.78; 95% CI, 0.61–0.99; p = 0.0238); PFS per RECIST 1.1 criteria was 3.0 vs. 2.8 months (HR, 0.71; 95% CI, 0.57 to 0.90; p = 0.0022). OS and PFS did not reach statistical significance according to pre-specified criteria (p = 0.0174 for OS and p = 0.0020 for PFS). ORR was 18.2% for pembrolizumab and 4.4% for placebo. A higher OS than expected was reported for the placebo arm; contributed to the approval of two second-line drugs during trial accrual, regorafenib and nivolumab; and deemed as a cause of KEYNOTE-240 failure.

Other PD-1/PD-L1 inhibitors tested as monotherapy in advanced HCC include durvalumab, cemiplimab, camrelizumab, and tislelizumab (Table 1).

**Table 1:** Main trials of ICIs for advanced HCC

Name	Drugs	Setting	Phase (n)	Results
CheckMate-040 NCT02828124	Nivolumab From 0.1 to 10 mg/kg Q2W (Dose escalation) 3 mg/kg Q2W (Dose expansion)	II line after sorafenib failure/intolerance	I/II dose escalation (48) Dose expansion (214)	ORR (mRECIST): 15% (dose-escalation group); 20% (dose-expansion group) mOS: 15.1 months FDA-approved 2017 II line in CPS-A or -B after sorafenib
CheckMate-459 NCT02576509	Nivolumab 240 mg Q2W vs. sorafenib 400 mg bid	I line	III open-label Nivolumab (371) Sorafenib (372)	mOS: 16.8 vs. 14.7 months (p: 0.07) ORR: 15%
CheckMate-040 Cohort 4 NCT01658878	Nivolumab 1 mg/kg (arm A) or 3 mg/kg (arm B) Q3W Plus ipilimumab 3 mg/kg (arm A) or 1 mg/kg (arm B), Q3W × 4 followed by nivolumab 240 mg Q2W or nivolumab 3 mg/kg Q2W plus ipilimumab 1 mg/kg Q6W (arm C)	II line after sorafenib failure/intolerance	I/II Arm A (50) Arm B (49) Arm C (49)	ORR (mRECIST): 31% (Arm A): 27% (Arm B): 29% (Arm C): mOS: 22.8 months (arm A); 12.5 months (arm B); 12.7 months (arm C) FDA-approved 2020 II line in CPS-A after sorafenib
CheckMate-9DW NCT04039607	Nivolumab 1 mg/kg Q3W plus ipilimumab 3 mg/kg Q3W × 4 followed by nivolumab 240 mg Q2W vs. lenvatinib 12 or 8 mg qd or sorafenib 400 mg bid	I line	III open-label (650) 1:1	Pending Primary endpoint: OS Completion date: September 2023
NCT04393220	Nivolumab 480 mg Q4W plus bevacizumab 15 mg/kg Q3W	I line	II open-label (60)	Pending Primary endpoints: PFS and OS Completion date: October 2021
Keynote-224 NCT02702414	Pembrolizumab 200 mg Q3W	II line after sorafenib failure/intolerance	II (104)	ORR (mRECIST): 17%; mPFS: 4.9 months; mOS: 12.9 months FDA-approved 2018 II line in CPS-A after sorafenib
Keynote-240 NCT02702401	Pembrolizumab 200 mg Q3W vs. placebo	II line after sorafenib failure/intolerance	III double-blind Nivolumab (278) Placebo (135)	mOS: 13.9 vs. 10.6 months (p: 0.02); mPFS: 3 vs. 2.8 months (p: 0.002)
Keynote-524 NCT03006926	Pembrolizumab Q3W plus lenvatinib 12 or 8 mg qd	I line	Ib (104)	ORR (mRECIST): 46%; mPFS: 9.3 months; mOS: 22 months
LEAP-002 NCT03713593	Pembrolizumab Q3W plus lenvatinib 12 or 8 mg qd vs. lenvatinib 12 or 8 mg qd plus placebo Q3W	I line	III double-blind (750) 1:1	Pending Primary endpoints: OS and PFS Completion date: May 2023
NCT02715531	Atezolizumab 1200 mg Q3W plus bevacizumab 15 mg/kg Q3W or atezolizumab 1200 mg Q3W	I line	Ib Atezo + Beva (60) Atezo (59)	mPFS: 5.6 vs. 3.4 months; ORR (mRECIST): 27% vs. 17% mOS not reached
IMbrave-150 NCT03434379	Atezolizumab 1200 mg Q3W plus bevacizumab 15 mg/kg Q3W vs. sorafenib 400 mg bid	I line	III open-label Atezo + Beva (336) sorafenib (165) 2:1	mPFS: 6.8 vs. 4.3 months; ORR: 27.3% vs. 11.9%; OS rate 6 and 12 months: 84.8% and 67.2% vs. 72.2% and 54.6% FDA-approved 2020 I line CPS-A
COSMIC-312 NCT03755791	Atezolizumab 1200 mg Q3W plus cabozantinib 40 mg qd vs. cabozantinib 60 mg qd vs. sorafenib 400 mg bid	I line	III open-label (740) 2:1:1	Pending Primary endpoints: OS and PFS Completion date: December 2021
NCT01693562	Durvalumab 10 mg/kg Q2W	II line after sorafenib failure/intolerance	I/II (40)	ORR: 10.3%; mOS: 13.2 months
NCT02519348	Tremelimumab 300 mg plus durvalumab 1500 mg 1 dose followed by durvalumab Q4W or tremelimumab 75 mg Q4W plus durvalumab 1500 mg Q4W × 4 doses followed by durvalumab Q4W or durvalumab 1500 mg Q4W or tremelimumab 750 mg Q4W	II line after sorafenib failure/intolerance	II T 300 + D (75) T 75 + D (84) D (104) T (69)	mOS: 18.7; 11.3; 11.7; 17.1 months; ORR: 22.7%; 11.3%; 11.7%; 17.1%
HIMALAYA NCT03298451	Durvalumab 1500 mg Q4W or tremelimumab 300 mg plus durvalumab 1500 mg 1 dose followed by durvalumab Q4W or tremelimumab 75 mg Q4W plus durvalumab 1500 mg Q4W × 4 doses followed by durvalumab Q4W or sorafenib 400 mg bid		III open-label (1324) 1:1:1:1	Pending Primary endpoint: OS Completion date: May 2021
NCT02572687	Durvalumab 750 mg Q2W plus ramucirumab 8 mg/kg Q2W	II line after sorafenib failure/intolerance	Ia/b (28)	mPFS: 4.4 months mOS: 10.7 months
VEGF Liver 100 NCT03289533	Avelumab 10 mg/kg Q2W plus axitinib 5 mg orally BID	I line	Ib (22)	ORR (mRECIST): 31.8% mPFS (mRECIST) 3.8 months
NCT02989922	Camrelizumab 3 mg/kg Q2W or Q3W	II line after previous treatment failure/intolerance	II Q3W (108) Q2W (109)	ORR: 14.7% OS rate at 6 months 74.7%
NCT03463876	Camrelizumab 200 mg Q2W plus apatinib 125–500 mg qd	II line after previous treatment failure/intolerance	Ia/b Dose escalation (15) Dose expansion (28)	ORR (RECIST): 30%; mPFS: 5.8 months; mOS: NR
NCT02407990	Tislelizumab 5 mg/kg Q3W	After at least 1 previous line (median: 2 previous therapies)	Ia/b (50)	ORR (RECIST) 12.2%
RATIONALE 301 NCT03412773	Tislelizumab 200 mg Q3W vs. sorafenib 400 mg bid	I line	III open-label (674)	Pending Primary endpoint: OS Completion date: May 2022
ORIENT-32 NCT03794440	Sintilimab 200 mg Q3W plus bevacizumab biosimilar 15 mg/kg Q3W vs. sorafenib 400 mg bid	I line (94%: HBV; 4.2%: CPS-B)	III open-label Sint + Beva (380) Sorafenib (191) 2:1	mOS: NR vs. 10.4 months; 43.1% reduced risk of death with Sint + Beva; mPFS (RECIST): 4.6 vs. 2.8 months ORR (RECIST): 20.5% vs. 4.1%
NCT04368078	Toripalimab 240 mg Q3W plus lenvatinib 12 or 8 mg qd	II line after I line progression with the exception of lenvatinib	II (76)	Pending Primary endpoint: ORR Completion date: April 2023

Durvalumab is a fully humanized IgG1 against PD-L1. In the expansion cohort of study NCT01693562 [17], a phase I/II trial of durvalumab in patients with advanced malignancies, 40 patients with advanced HCC were enrolled. Grade 3 or higher AEs were reported in 20% of patients. ORR by RECIST 1.1 was 10.3% in the overall population and 25% in patients affected by HCV (8) translating in a median OS of 13.2 was reported in the overall population and 19.3 months in the HCV population [18]. The reasons for the better efficacy of durvalumab in HCV patients have not been elucidated; in fact, HCC etiology does not seem to influence activity of other ICIs.

Tislelizumab (BGB-A317), a monoclonal IgG4 against PD-1, is currently being tested in a phase III non-inferiority trial (RATIONALE 301) against sorafenib [19].

Less mature data are available for cemiplimab, another anti PD-1 antibody (REGN2810) [20].

## CTLA-4 inhibitors

CTLA-4, expressed by regulatory and activated T-cells, transmits inhibitory signals to effector T-cells on interaction with B7-1 and B7-2 expressed on the membrane of antigen-presenting cells. CTLA-4 competes with T-cell stimulatory CD28 in this interaction and thereby functions as another immune checkpoint molecule [21]. CTLA-4 blockade mediates anti-tumoural activity by suppressing this inhibitory interaction, enhancing cytotoxic T-cell activity [22]. Ipilimumab was the first ICI approved in 2010 [22]. Its efficacy is significantly enhanced by the combination with other ICIs in a broad range of tumours [23]. Nowadays, the use of CTLA-4 inhibitors is mainly conceived in association with PD-1-directed antibodies.

Tremelimumab, a fully humanized IgG2, was the first CTLA-4 inhibitor tested as monotherapy in HCC [24]. A cohort of 20 HCV-positive patients with HCC received 3 mg/kg of tremelimumab every 90 days until disease progression or toxicity. The safety profile was acceptable; grade 2 or higher transaminase elevation was the most common side effect. In total, 17.6% of the patients had a partial response and the study reported a TTP of 6.5 months. Interestingly, a significant drop in HCV RNA was observed [24]. More recently, tremelimumab was tested in association with subtotal radiofrequency ablation, chemoablation, or trans-arterial chemoembolization in patients with advanced HCC. The rationale was to exploit both local and abscopal effect of locoregional treatments to enhance the response to ICIs [25]. Median TTP was 7.4 months, median OS was 12.3 months, and 26% of patients had a partial response. In keeping with previous data [24], 12 of 14 patients with quantifiable HCV RNA at study enrolment had reduction in viral load whilst on tremelimumab [25].

## Dual checkpoint inhibition

Inhibition of both PD-1 and CTLA-4 pathways is known to give superior outcomes compared to monotherapy across several malignancies. Combined therapy is currently approved for melanoma [26], renal cell carcinoma [27], non-small cell lung carcinoma [28], microsatellite instability-high colorectal cancer [29], and advanced HCC [30•].

CheckMate-040 later incorporated a dual checkpoint inhibition arm with nivolumab and ipilimumab [30•]. In total, 148 CPS-A and ECOG PS 0-1 patients were randomized 1:1:1, to receive nivolumab and ipilimumab at different doses. Safety, tolerability, and ORR were primary endpoints. PD-L1 status was evaluated using the 28-8 pharmDx assay. The highest ORR was seen in arm A of the study, wherein 50 patients were treated with nivolumab 1 mg/kg plus ipilimumab 3 mg/kg every 3 weeks for 4 doses, followed by nivolumab 240 mg every 2 weeks. ORR was 32% vs. 27% in arm B and 29% in arm C, and median OS was 22.8 months (vs. 12.5 and 12.7 in arms B and C). Responses were independent of PD-L1 expression and HCC etiology [30•]. Patients in arm A experienced a higher rate of AEs of any grade (94% vs. 71% vs. 79%); however, the types of AE were similar among the 3 arms and consistent with previous studies. Albeit at the expense of increased toxicity, the combination of ipilimumab and nivolumab gave the longest OS ever observed in HCC patients in a second-line setting. In the wake of these results, the FD-approved combination ipilimumab and nivolumab in the arm A dosing regimen as second-line therapy for patients with preserved liver function (CP A), acknowledging the compromise between treatment efficacy and adverse effects.

Currently, the same combination is under investigation in a phase III trial (CheckMate-9DW) as a first-line treatment for advanced HCC [31] (Table 1).

Durvalumab plus tremelimumab is another ICI combination of interest in HCC. Safety and efficacy of the combination has been tested in a phase I/II trial [32]. The study enrolled 332 patients intolerant or resistant to sorafenib. Participants were randomized to receive the combination of durvalumab plus tremelimumab with two different schedules, or monotherapy with durvalumab or tremelimumab. The combination of tremelimumab at 300 mg (T300) single dose plus durvalumab at 1500 mg every 4 weeks achieved the best ORR: 24%, and median OS: 18.7 months. PFS did not differ across the regimens. In the T300 arm, 16% of patients had serious TRAE, interestingly lower than in tremelimumab monotherapy (25%). No differences according to HCC etiology were reported. Tremelimumab and durvalumab dual therapy has since progressed to phase III investigation, as front-line treatment (HIMALAYA study) [33]. The trial has completed the accrual phase with results pending.

After the approval of ipilimumab plus nivolumab as a second-line therapy, dual checkpoint inhibition is expected to transform the first-line landscape [30•]. A major limitation of this strategy is inaccessibility to numerous HCC patients with reduced liver function and performance status; phase I/II safety testing in these patients warrants consideration.

## PD-1/PD-L1 plus VEGF inhibitors

The rationale of combining anti PD-1 drugs with anti-angiogenic agents has been well-established [34]. Data from preclinical models showed that vascular endothelial growth factor (VEGF) expression associates with reduced T-cell activity; conversely, anti-VEGF therapy increased abundance of tumour-infiltrating lymphocytes, possibly through endothelial stabilization [34].

In HCC, the first evidence of efficacy emerged from a phase Ib trial of combined atezolizumab (IgG1 anti PD-L1) and bevacizumab (anti-VEGF-A) in previously untreated advanced HCC [35]. Patients treated with dual atezolizumab and bevacizumab had an ORR of 36% and median PFS of 5.6 months. Adverse events were consistent with the known profiles of the two drugs. The most common grade 3 or 4 AEs were hypertension (13%) and proteinuria (7%). Serious TRAEs occurred in 24% of participants.

IMbrave-150, a phase III, open-label randomized trial, recently confirmed the efficacy of this combination as a front-line therapy [36••]. Overall, 336 patients with histologically or cytologically confirmed advanced HCC not amenable for curative surgery or radical locoregional treatments, CPS-A, ECOG PS 0-1, naïve from systemic therapies, were randomized with a 2:1 ratio to receive atezolizumab 1200-mg flat dose plus bevacizumab 15 mg/kg every 3 weeks, or sorafenib 400 mg bid. OS and PFS in the ITT population were coprimary endpoints. At the time of data cut-off (August 2019), median PFS was significantly longer for the combination of atezolizumab and bevacizumab, 6.8 months vs. 4.3 months for sorafenib, with a HR of 0.59 (95% CI: 0.4–0.76; p < 0.001). ORR assessed by RECIST 1.1 was 27.3% for the combination and 11.9% for sorafenib. Serious AEs were reported to have higher incidence in the combination group (38% vs. 30%), most commonly hypertension (15.2%), in keeping with the known profile for bevacizumab. On the other hand, patients in the sorafenib arm had higher incidence of AEs more severely affecting quality of life, such as diarrhoea, decreased appetite, and palmar-plantar erythrodysesthesia.

The combination of atezolizumab and bevacizumab was the first front-line therapy to achieve an OS superior to sorafenib, which led to approval by the FDA [37]. Updated results have been published for IMbrave-150: overall survival in the combination group was 19.2 months vs. 13.4 with sorafenib (HR 0.66, 95% CI: 0.52–0.85, p = 0.0009) [38].

A similar approach was adopted in the Chinese phase II/III ORIENT-32 trial [39]. The study randomized 566 patients naïve from systemic treatment to receive the combination of sintilimab (anti PD-1 antibody) plus bevacizumab biosimilar (380) or sorafenib (191) in a 2:1 ratio. The experimental arm recorded higher OS and PFS; primary endpoints were met.

A number of other trials are investigating other combinations of ICI plus VEGF inhibitors.

The combination of nivolumab and bevacizumab is currently under investigation for a first-line treatment of advanced HCC in a phase II trial (Table 1) [40].

Recently, the combination of durvalumab and ramucirumab has been tested in a phase Ia/b multicohort trial [41]. HCC patients achieved an ORR of 11%; median PFS and median OS were 4.4 and 10.7 months, respectively (Table 1).

## PD-1/PD-L1 plus multi-targeted tyrosine kinase inhibitors

Tyrosine kinase inhibitors (TKIs) have been the cornerstone of HCC treatment for many years [42]. Since the approval of sorafenib in 2007 [13], many molecular targeted therapies have been tested with varying degrees of success [13]. The approval of nivolumab in 2017 [9] despite negative phase III trials invited combination ICI and TKI as a therapeutic approach. Synergistic anti-tumour activity has been demonstrated in preclinical models [43]. TKIs act by blocking several angiogenic pathways [44] with consequent stabilization of vascular endothelia in the tumour bed [2]. Exposure to TKIs has been reported to increase inflammatory cell infiltrate. Furthermore, resistance to anti-angiogenic drugs is known to be related to immunosuppressive microenvironment, sustained by increased T-reg activity and PD-L1 overexpression [45]. These observations strengthen the rationale of combining TKIs with immune therapies [45].

The combination of pembrolizumab and lenvatinib (a multi-TKI inhibitor, non-inferior to sorafenib, approved as a first-line treatment [46•]) was investigated in Keynote-524, a phase Ib trial [47]. The dose-limiting toxicity (DLT) phase was carried out with a 3 + 3 design; no DLTs emerged. In total, 100 CPS-A patients were enrolled in the expansion phase, receiving 200 mg of pembrolizumab every 3 weeks and 12 mg (if body weight ≥ 60 kg) or 8 mg (if bodyweight < 60 kg) of lenvatinib daily. Patients with bile duct or main trunk portal vein invasion were excluded. Primary endpoints of dose-expansion phase were ORR and duration of response (DOR) by RECIST 1.1 and by modified RECIST (mRECIST) criteria per independent imaging review. ORR was 46% by mRECIST and 36% by RECIST 1.1 criteria. Median DOR was 8.6 months by mRECIST and 12.6 months by RECIST 1.1. Median PFS was 9.3 months and median OS 22.0 months. At the end of the study, 99% of the patients had at least 1 AE. Overall, 67% of the patients reported grade 3 or higher TRAEs, the most frequent being hypertension (17%). Notably, grade 5 AEs occurred in 13 patients (13%), 3 deemed treatment-related [48]. In July 2019, the FDA granted breakthrough therapy designation for the combination based on an interim results analysis of the study. Accelerated approval however declined the following year, with the FDA having just approved the combination of atezolizumab and bevacizumab.

The same combination is being investigated in a phase III open-label trial (LEAP-002) against lenvatinib monotherapy as a first-line treatment for advanced HCC. The trial enrolled 750 patients randomized 1:1. OS and PFS are the primary endpoints. Patients are stratified according to macroscopic vascular invasion, metastatic disease, ECOG PS (0–1), and AFP level (≤ 400 or > 400 ng/mL). The trial has recently completed the accrual phase; results are still pending [49].

COSMIC-312 is another phase III randomized trial that is assessing the association of atezolizumab and cabozantinib. Cabozantinib is an oral TKI that inhibits, among others, VEGF receptors, c-Met, and AXL. The study has 3 arms: patients with advanced HCC not amenable to surgery or locoregional treatments and have not received prior systemic therapies are randomized 2:1:1 to receive atezolizumab (1200 mg every 3 weeks) plus cabozantinib 40 mg daily or cabozantinib 60 mg daily, or sorafenib 400 mg bid. OS and PFS are primary endpoints. The trial is currently recruiting [50].

Avelumab (anti PD-L1 IgG1 antibody) plus axitinib has been studied with a similar rationale [51]. VEGF Liver 100 was a phase Ib trial that assessed the safety and activity of combination avelumab 10 mg/kg every 2 weeks and axitinib 5 mg qd as a first-line therapy for advanced HCC. At data cut-off, 22 patients were treated, ORR according to RECIST 1.1 was 13.6% (31.8% per mRECIST), and median PFS was 5.5 months per RECIST 1.1. No grade 4 or 5 TRAEs were recorded, the most common grade 3 treatment-related AEs were hypertension (50%) and hand foot syndrome (22.7%). Similar results have been reported for the combination of camrelizumab (anti PD-1 antibody) and apatinib (anti VEGFR-2) in a phase II trial (RESCUE) (Table 1) [52]. The phase I/II CheckMate-040 trial has been further developed to include a cohort treated with ipilimumab, nivolumab, and cabozantinib triple therapy [53]. In this arm, 70 sorafenib-experienced or naive patients were randomized 1:1 to receive nivolumab (240 mg every 2 weeks) plus cabozantinib (40 mg qd) or these two drugs at the same dose plus ipilimumab (1 mg/kg every 6 weeks). ORR was 17% for patients treated with nivolumab and cabozantinib, and 26% for the triplet arm. Median PFS was 5.5 months vs. 6.8 months in the doublet and triplet arm, respectively. Seventeen percent of the patients treated with nivolumab plus ipilimumab plus cabozantinib had grade 3 or higher AEs, compared to 42% in the other arm [53]. An ongoing phase II trial is testing the combination of toripalimab (anti PD-1) and lenvatinib in a first-line refractory HCC [54].

With an emerging growth of therapeutic options for advanced HCC, identification of predictive biomarkers is a highly unmet need to optimize treatment to the individual patient and avoid unnecessary toxicity.

## Adjuvant and neoadjuvant immunotherapy

Whilst tumour resection, OLT, and local ablation are potentially curative for HCC (as well as underlying liver disease in the case of OLT), most patients are unfortunately diagnosed at an advanced stage wherein disease in unamenable to radical treatments [55]. Furthermore, recurrence rate can be as high as 70% after resection and 10% after OLT [2]. Effective neoadjuvant therapies are required to increase the number of patients eligible for curative therapies, and to improve post-operative outcomes. Similarly, effective adjuvant therapies are needed to reduce the risk of early and late recurrences after curative treatments (resection or ablation). No adjuvant or neoadjuvant treatments are currently licensed for treatment of HCC [1].

As adjuvant treatment, sorafenib failed to show advantages in term of relapse-free survival (RFS) after radical resection or ablation in a large phase III trial [56]. Immunotherapy has also been trialled in these settings, with several phase III trials currently recruiting patients after surgical resection or ablation (Table 2).

**Table 2:** Main trials of ICIs in adjuvant and neoadjuvant setting

Name	Drugs	Setting	Phase	Results
CheckMate-9DX NCT03383458	Nivolumab vs. placebo	Adjuvant after curative resection or ablation	III	Pending Completion date: June 2025
KEYNOTE-937 NCT03867084	Pembrolizumab vs. placebo	Adjuvant after curative resection or ablation	III	Pending Completion date: June 2025
JUPITER 04 NCT03859128	Toripalimab	Adjuvant after curative resection	II/III	Pending Completion date: April 2024
NCT04639180	Camrelizumab plus apatinib	Adjuvant after curative resection or ablation	III	Pending Completion date: July 2024
EMERALD-2 NCT03847428	Durvalumab plus bevacizumab or durvalumab vs. placebo	Adjuvant after curative resection or ablation	III	Pending Completion date: September 2023
IMbrave-050 NCT04102098	Atezolizumab plus bevacizumab	Adjuvant after curative resection or ablation		Pending Completion date: August 2027
NCT04615143	Tislelizumab	Neoadjuvant for resectable recurrent HCC	II	Pending Completion date: June 2022
NCT03916627	Cemiplimab	Neoadjuvant for Resectable HCC	II	Pending Completion date: August 2027
NCT03867370	Toripalimab	Neoadjuvant for resectable HCC or ICC	I/II	Pending Completion date: November 2021
NIVOLEP NCT03630640	Nivolumab	Neoadjuvant in patients eligible for electroporation (single nodule>3 cm < 5 cm, multinodular) adjuvant after electroporation	II	Pending Completion date: September 2020
NCT04123379	Nivolumab plus anti-IL-8 or oral CCR2/5-inhibitor	Neoadjuvant for resectable HCC and adjuvant after surgery	II	Pending Completion date: October 2024
NCT03510871	Ipilimumab plus nivolumab	Neoadjuvant for HCC potentially eligible for curative surgery	II	Pending Completion date: December 2022
PRIME-HCC NCT03682276	Ipilimumab plus nivolumab	Neoadjuvant for resectable HCC	Ib	Pending Completion date: September 2022
NCT04297202	SHR-1210 (anti PD-1) plus apatinib	Neoadjuvant for resectable HCC and adjuvant treatment after surgery	II	Pending Completion date: December 2021

Cemiplimab [57], toripalimab [58], and nivolumab [59] are among the ICIs currently under investigation as neoadjuvant treatments (Table 2). The combination of ipilimumab and nivolumab is also being tested in a phase II trial [60] and a further phase 1b study, PRIME-HCC, incorporating tumour and stool biomarker analysis [61]. The paradigm of neoadjuvant therapy prior to organ transplantation is perhaps unique to the treatment of liver cancer, but certainly plausible. Currently, there are not enough data to recommend the use of ICIs before or after OLT in clinical practice. Data from large clinical trials will be invaluable to define the safety and feasibility of this approach. In fact, the anti-cancer effect of ICIs should be carefully balanced against the intrinsic risk of organ rejection.

## Immunotherapy plus locoregional treatments

Despite efforts to improve surveillance strategies in patients at risk, about 60% of HCCs are diagnosed at intermediate-stage (BCLC-B) and are non-amenable for surgery or ablation [62]. The standard of care for BCLC-B HCC remains palliative trans-arterial chemoembolization (TACE), independent of tumour extension [63]*.* The combination of TACE with sorafenib has been evaluated in a placebo-controlled trial, but with no significant change in TTP [64]. TACE-induced tumour ischaemia triggers hypoxia inducible factor-1α (HIF) expression [65], which is known to associate with PD-L1 expression [66]. Inferentially, TACE may therefore potentiate the efficacy of PD-1/PD-L1 inhibitors. T regulatory cell populations (of total CD4-positive T lymphocytes) are higher in HCC patients vs. healthy controls, but decrease after TACE. Moreover, CD4+/CD8+ ratio is significantly lower in HCC patients and markedly increases after TACE [67]. These changes in immune cell populations infer a potential therapeutic niche for ICIs after TACE. Data from phase III trials are currently lacking; however, this combination is being investigated in phase II trials.

Results from these early trials could begin to significantly advance the treatment of intermediate-stage HCC. Given the heterogeneity of intermediate-stage disease, however, development of prognostic stratification is likely to be important for future trial design and clinical practice.

## Conclusions

Immunotherapy, in PD-1/PD-L1 inhibitors, CTLA4 inhibitors, and beyond, shows transforming potential for treatment of HCC, particularly in combination regimens. Identifying the agents and combinations which balance potency and risk in patients with different HCC stages, degree of underlying liver disease, and performance status should be the priority for ongoing clinical trials. Establishing utility in the adjuvant and neoadjuvant setting is needed to improve long-term survival in HCC, whilst assessing safety in patients with poor liver function and advanced disease is needed to improve OS for this more deprived patient group. HCC etiology seems to influence outcome only in the case of durvalumab monotherapy; however, this aspect should be further investigated. Finally, predictive biomarkers for response beyond the limited role for PD-L1 expression are greatly needed to tailor HCC immunotherapy.

## References

[1] Tovoli F, De Lorenzo S, Trevisani F. Immunotherapy with checkpoint inhibitors for hepatocellular carcinoma: where are we now? Vaccines (Basel). 2020;8(4). 10.3390/vaccines8040578. 10.3390/vaccines8040578 PMC7711845 33023131

[2] Flynn MJ, Sayed AA, Sharma R, Siddique A, Pinato DJ (2019). Challenges and opportunities in the clinical development of immune checkpoint inhibitors for hepatocellular carcinoma. Hepatology..

[3] Pardoll DM (2012). The blockade of immune checkpoints in cancer immunotherapy. Nat Rev Cancer.

[4] Kudo M (2017). Immuno-oncology in hepatocellular carcinoma: 2017 update. Oncology..

[5] Mima K, Nakagawa S, Sawayama H, Ishimoto T, Imai K, Iwatsuki M (2017). The microbiome and hepatobiliary-pancreatic cancers. Cancer Lett.

[6] Wang BJ, Bao JJ, Wang JZ, Wang Y, Jiang M, Xing MY (2011). Immunostaining of PD-1/PD-Ls in liver tissues of patients with hepatitis and hepatocellular carcinoma. World J Gastroenterol.

[7] Brown ZJ, Heinrich B, Steinberg SM, Yu SJ, Greten TF (2017). Safety in treatment of hepatocellular carcinoma with immune checkpoint inhibitors as compared to melanoma and non-small cell lung cancer. J Immunother Cancer.

[8] El-Khoueiry AB, Sangro B, Yau T, Crocenzi TS, Kudo M, Hsu C (2017). Nivolumab in patients with advanced hepatocellular carcinoma (CheckMate 040): an open-label, non-comparative, phase 1/2 dose escalation and expansion trial. Lancet..

[9] Finkelmeier F, Waidmann O, Trojan J (2018). Nivolumab for the treatment of hepatocellular carcinoma. Expert Rev Anticancer Ther.

[10] El-Khoueiry AB, Melero I, Yau TC, Crocenzi TS, Kudo M, Hsu C (2018). Impact of antitumor activity on survival outcomes, and nonconventional benefit, with nivolumab (NIVO) in patients with advanced hepatocellular carcinoma (aHCC): subanalyses of CheckMate-040. J Clin Oncol.

[11] An investigational immuno-therapy study of nivolumab compared to sorafenib as a first treatment in patients with advanced hepatocellular carcinoma. https://ClinicalTrials.gov/show/NCT02576509.

[12] Yau T, Park JW, Finn RS, Cheng AL, Mathurin P, Edeline J (2019). LBA38_PR - CheckMate 459: a randomized, multi-center phase III study of nivolumab (NIVO) vs sorafenib (SOR) as first-line (1L) treatment in patients (pts) with advanced hepatocellular carcinoma (aHCC). Ann Oncol.

[13] Llovet JM, Ricci S, Mazzaferro V, Hilgard P, Gane E, Blanc JF (2008). Sorafenib in advanced hepatocellular carcinoma. N Engl J Med.

[14] Zhu AX, Finn RS, Edeline J, Cattan S, Ogasawara S, Palmer D (2018). Pembrolizumab in patients with advanced hepatocellular carcinoma previously treated with sorafenib (KEYNOTE-224): a non-randomised, open-label phase 2 trial. Lancet Oncol.

[15] Finn RS, Ryoo BY, Merle P, Kudo M, Bouattour M, Lim HY (2020). Pembrolizumab as second-line therapy in patients with advanced hepatocellular carcinoma in KEYNOTE-240: a randomized, double-blind, phase III trial. J Clin Oncol.

[16] Dolled-Filhart M, Roach C, Toland G, Stanforth D, Jansson M, Lubiniecki GM (2016). Development of a companion diagnostic for pembrolizumab in non-small cell lung cancer using immunohistochemistry for programmed death ligand-1. Arch Pathol Lab Med.

[17] A phase 1/2 study to evaluate MEDI4736. https://ClinicalTrials.gov/show/NCT01693562.

[18] Wainberg ZA, Segal NH, Jaeger D, Lee K-H, Marshall J, Antonia SJ (2017). Safety and clinical activity of durvalumab monotherapy in patients with hepatocellular carcinoma (HCC). J Clin Oncol.

[19] Phase 3 study of tislelizumab versus sorafenib in participants with unresectable HCC. https://ClinicalTrials.gov/show/NCT03412773.

[20] Migden MR, Rischin D, Schmults CD, Guminski A, Hauschild A, Lewis KD (2018). PD-1 blockade with cemiplimab in advanced cutaneous squamous-cell carcinoma. N Engl J Med.

[21] Chan LL, Chan SL. Emerging immune checkpoint inhibitors for the treatment of hepatocellular carcinoma. Expert Opin Emerg Drugs. 2021:1–13. 10.1080/14728214.2021.1902503. 10.1080/14728214.2021.1902503 33724135

[22] Hodi FS, O'Day SJ, McDermott DF, Weber RW, Sosman JA, Haanen JB (2010). Improved survival with ipilimumab in patients with metastatic melanoma. N Engl J Med.

[23] Xing P, Zhang F, Wang G, Xu Y, Li C, Wang S (2019). Incidence rates of immune-related adverse events and their correlation with response in advanced solid tumours treated with NIVO or NIVO+IPI: a systematic review and meta-analysis. J Immunother Cancer.

[24] Sangro B, Gomez-Martin C, de la Mata M, Inarrairaegui M, Garralda E, Barrera P (2013). A clinical trial of CTLA-4 blockade with tremelimumab in patients with hepatocellular carcinoma and chronic hepatitis C. J Hepatol.

[25] Duffy AG, Ulahannan SV, Makorova-Rusher O, Rahma O, Wedemeyer H, Pratt D (2017). Tremelimumab in combination with ablation in patients with advanced hepatocellular carcinoma. J Hepatol.

[26] Larkin J, Chiarion-Sileni V, Gonzalez R, Grob JJ, Cowey CL, Lao CD (2015). Combined nivolumab and ipilimumab or monotherapy in untreated melanoma. N Engl J Med.

[27] Albiges L, Tannir NM, Burotto M, McDermott D, Plimack ER, Barthelemy P (2020). Nivolumab plus ipilimumab versus sunitinib for first-line treatment of advanced renal cell carcinoma: extended 4-year follow-up of the phase III CheckMate 214 trial. ESMO Open.

[28] Hellmann MD, Paz-Ares L, Bernabe Caro R, Zurawski B, Kim SW, Carcereny Costa E (2019). Nivolumab plus ipilimumab in advanced non-small-cell lung cancer. N Engl J Med.

[29] Overman MJ, Lonardi S, Wong KYM, Lenz HJ, Gelsomino F, Aglietta M (2018). Durable clinical benefit with nivolumab plus ipilimumab in DNA mismatch repair-deficient/microsatellite instability-high metastatic colorectal cancer. J Clin Oncol.

[30.•] Yau T, Kang YK, Kim TY, El-Khoueiry AB, Santoro A, Sangro B, et al. Efficacy and safety of nivolumab plus ipilimumab in patients with advanced hepatocellular carcinoma previously treated with sorafenib: the CheckMate 040 randomized clinical trial. JAMA Oncol. 2020;6(11):e204564. 10.1001/jamaoncol.2020.4564 Phase I/II trial resulting in FDA approval of ipilimumab plus nivolumab in advanced HCC after sorafenib. 10.1001/jamaoncol.2020.4564 PMC7530824 33001135

[31] A study of nivolumab in combination with ipilimumab in participants with advanced hepatocellular carcinoma. https://ClinicalTrials.gov/show/NCT04039607.

[32] Kelley R, Kudo M, Harris W, Ikeda M, Okusaka T, Kang Y (2020). O-6 The novel regimen of tremelimumab in combination with durvalumab provides a favorable safety profile and clinical activity for patients with advanced hepatocellular carcinoma. Ann Oncol.

[33] Kelley RK, Sangro B, Harris WP, Ikeda M, Okusaka T, Kang Y-K (2020). Efficacy, tolerability, and biologic activity of a novel regimen of tremelimumab (T) in combination with durvalumab (D) for patients (pts) with advanced hepatocellular carcinoma (aHCC). J Clin Oncol.

[34] Wallin JJ, Bendell JC, Funke R, Sznol M, Korski K, Jones S (2016). Atezolizumab in combination with bevacizumab enhances antigen-specific T-cell migration in metastatic renal cell carcinoma. Nat Commun.

[35] Lee MS, Ryoo BY, Hsu CH, Numata K, Stein S, Verret W (2020). Atezolizumab with or without bevacizumab in unresectable hepatocellular carcinoma (GO30140): an open-label, multicentre, phase 1b study. Lancet Oncol.

[36] Finn RS, Qin S, Ikeda M, Galle PR, Ducreux M, Kim TY (2020). Atezolizumab plus bevacizumab in unresectable hepatocellular carcinoma. N Engl J Med.

[37] Casak SJ, Donoghue M, Fashoyin-Aje L, Jiang X, Rodriguez L, Shen YL, et al. FDA Approval summary: atezolizumab plus bevacizumab for the treatment of patients with advanced unresectable or metastatic hepatocellular carcinoma. Clin Cancer Res. 2020. 10.1158/1078-0432.CCR-20-3407. 10.1158/1078-0432.CCR-20-3407 33139264

[38] Finn RS, Qin S, Ikeda M, Galle PR, Ducreux M, Kim T-Y (2021). IMbrave150: Updated overall survival (OS) data from a global, randomized, open-label phase III study of atezolizumab (atezo) + bevacizumab (bev) versus sorafenib (sor) in patients (pts) with unresectable hepatocellular carcinoma (HCC). J Clin Oncol.

[39] A study to evaluate the efficacy and safety of sintilimab in combination with IBI305 (anti-VEGF monoclonal antibody) compared to sorafenib as the first-line treatment for advanced hepatocellular carcinoma. https://ClinicalTrials.gov/show/NCT03794440

[40] Combination of PD-1 and VEGFR-2 blockade for advanced hepatocellular carcinoma. https://ClinicalTrials.gov/show/NCT04393220

[41] Bang YJ, Golan T, Dahan L, Fu S, Moreno V, Park K (2020). Ramucirumab and durvalumab for previously treated, advanced non-small-cell lung cancer, gastric/gastro-oesophageal junction adenocarcinoma, or hepatocellular carcinoma: an open-label, phase Ia/b study (JVDJ). Eur J Cancer.

[42] Liu Z, Lin Y, Zhang J, Zhang Y, Li Y, Liu Z (2019). Molecular targeted and immune checkpoint therapy for advanced hepatocellular carcinoma. J Exp Clin Cancer Res.

[43] Schaaf MB, Garg AD, Agostinis P (2018). Defining the role of the tumor vasculature in antitumor immunity and immunotherapy. Cell Death Dis.

[44] Wong CH, Wong CS, Chan SL (2015). Targeting angiogenic genes as a therapeutic approach for hepatocellular carcinoma. Curr Gene Ther.

[45] Pinato DJ, Guerra N, Fessas P, Murphy R, Mineo T, Mauri FA (2020). Immune-based therapies for hepatocellular carcinoma. Oncogene..

[46] Kudo M, Finn RS, Qin S, Han KH, Ikeda K, Piscaglia F (2018). Lenvatinib versus sorafenib in first-line treatment of patients with unresectable hepatocellular carcinoma: a randomised phase 3 non-inferiority trial. Lancet..

[47] A trial of lenvatinib plus pembrolizumab in participants with hepatocellular carcinoma. https://ClinicalTrials.gov/show/NCT03006926

[48] Finn RS, Ikeda M, Zhu AX, Sung MW, Baron AD, Kudo M (2020). Phase Ib study of lenvatinib plus pembrolizumab in patients with unresectable hepatocellular carcinoma. J Clin Oncol.

[49] Safety and efficacy of lenvatinib (E7080/MK-7902) in combination with pembrolizumab (MK-3475) versus lenvatinib as first-line therapy in participants with advanced hepatocellular carcinoma (MK-7902-002/E7080-G000-311/LEAP-002). https://ClinicalTrials.gov/show/NCT03713593

[50] Study of cabozantinib in combination with atezolizumab versus sorafenib in subjects with advanced HCC who have not received previous systemic anticancer therapy. https://ClinicalTrials.gov/show/NCT03755791

[51] A study of avelumab in combination with axitinib in advanced HCC (VEGF Liver 100). https://ClinicalTrials.gov/show/NCT03289533 10.1159/000514420 PMC8237783 34239811

[52] Xu J, Shen J, Gu S, Zhang Y, Wu L, Wu J, et al. Camrelizumab in combination with apatinib in patients with advanced hepatocellular carcinoma (RESCUE): a nonrandomized, open-label, phase II trial. Clin Cancer Res. 2020. 10.1158/1078-0432.CCR-20-2571. 10.1158/1078-0432.CCR-20-2571 33087333

[53] An immuno-therapy study to evaluate the effectiveness, safety and tolerability of nivolumab or nivolumab in combination with other agents in patients with advanced liver cancer. https://ClinicalTrials.gov/show/NCT01658878

[54] Lenvatinib combined toripalimab in advanced hepatocellular carcinoma. https://ClinicalTrials.gov/show/NCT04368078

[55] Akateh C, Black SM, Conteh L, Miller ED, Noonan A, Elliott E (2019). Neoadjuvant and adjuvant treatment strategies for hepatocellular carcinoma. World J Gastroenterol.

[56] Bruix J, Takayama T, Mazzaferro V, Chau GY, Yang J, Kudo M (2015). Adjuvant sorafenib for hepatocellular carcinoma after resection or ablation (STORM): a phase 3, randomised, double-blind, placebo-controlled trial. Lancet Oncol.

[57] Neoadjuvant cemiplimab for the treatment of resectable NSCLC, HCC, and HNSCC. https://ClinicalTrials.gov/show/NCT03916627

[58] Toripalimab or placebo as neoadjuvant therapy in resectable hepatocellular carcinoma or intrahepatic cholangiocarcinoma. https://ClinicalTrials.gov/show/NCT03867370

[59] Neoadjuvant and adjuvant nivolumab in HCC patients treated by electroporation. https://ClinicalTrials.gov/show/NCT03630640

[60] Nivolumab plus ipilimumab as neoadjuvant therapy for hepatocellular carcinoma (HCC). https://ClinicalTrials.gov/show/NCT03510871

[61] Safety and bioactivity of ipilimumab and nivolumab combination prior to liver resection in hepatocellular carcinoma. https://ClinicalTrials.gov/show/NCT03682276

[62] Dorn DP, Bryant MK, Zarzour J, Smith JK, Redden DT, Saddekni S (2014). Chemoembolization outcomes for hepatocellular carcinoma in cirrhotic patients with compromised liver function. HPB (Oxford).

[63] Pinato DJ, Howell J, Ramaswami R, Sharma R (2017). Review article: delivering precision oncology in intermediate-stage liver cancer. Aliment Pharmacol Ther.

[64] Lencioni R, Llovet JM, Han G, Tak WY, Yang J, Guglielmi A (2016). Sorafenib or placebo plus TACE with doxorubicin-eluting beads for intermediate stage HCC: The SPACE trial. J Hepatol.

[65] Liu K, Min XL, Peng J, Yang K, Yang L, Zhang XM (2016). The changes of HIF-1alpha and VEGF expression after TACE in patients with hepatocellular carcinoma. J Clin Med Res.

[66] Chen J, Jiang CC, Jin L, Zhang XD (2016). Regulation of PD-L1: a novel role of pro-survival signalling in cancer. Ann Oncol.

[67] Liao J, Xiao J, Zhou Y, Liu Z, Wang C (2015). Effect of transcatheter arterial chemoembolization on cellular immune function and regulatory T cells in patients with hepatocellular carcinoma. Mol Med Rep.

